# Supplementary material for: Circulating Galectin-3 in Patients with Cardiogenic Shock Complicating Acute Myocardial Infarction Treated with Mild Hypothermia: A Biomarker Sub-Study of the SHOCK-COOL Trial
Source: J Clin Med. 2022 Dec 2;11(23):7168. doi: 10.3390/jcm11237168 (PMC9740246; doi:10.3390/jcm11237168)
Supplement: Supplementary file 1 [file jcm-11-07168-s001.zip › jcm-2048373-supplementary.pdf]

Table S1. **Differences in Gal-3 levels between the MTH and Control groups on day 1, day 2, and day 3.**							
Gal-3 (ng/ml)	MTH vs. Control* Day 1	p-value	MTH vs. Control* Day 2	p-value	MTH vs. Control* Day 3	p-value	P for interaction &
Before imputation	-1.34 (-4.67 to 1.14)	0.223	-0.37 (-2.50 to 1.26)	0.532	0.27 (-1.31 to 1.74)	0.770	0.242
After imputation 1	-0.70 (-3.37 to 1.75)	0.380	-0.37 (-2.30 to 1.16)	0.497	0.16 (-0.69 to 1.40)	0.836	0.240
After imputation 2	-0.75 (-3.88 to 1.49)	0.310	0.19 (-1.24 to 1.50)	0.846	0.96 (-0.36 to 2.06)	0.208	0.209
After imputation 3	-1.02 (-3.42 to 1.72)	0.324	-0.11 (-1.55 to 1.16)	0.597	0.43 (-0.62 to 1.53)	0.425	0.192
After imputation 4	-0.61 (-3.37 to 1.87)	0.470	-0.76 (-2.50 to 0.56)	0.220	-0.30 (-1.08 to 0.91)	0.653	0.327
After imputation 5	-0.61 (-3.37 to 1.87)	0.474	-0.14 (-1.8 to 1.23)	0.774	0.40 (-0.56 to 1.55)	0.524	0.214

* Results are expressed as median difference and 95% confidence interval. & Interaction of Gal-3 in the MTH and Control groups over time.
MTH, Mild therapeutic hypothermia. Gal-3, galectin-3.

Table S2. Multiple comparisons of Gal-3 differences within the MTH and Control groups during three days of hospitalization.

	MTH Group						Control Group					
Gal-3 (ng/ml)	Day 1 Median (IQR)	Day 2 Median (IQR)	Day3 Median (IQR)	Dunn's test*			Day 1 Median (IQR)	Day 2 Median (IQR)	Day3 Median (IQR)	Dunn's test*		
				Day 1 vs 2	Day 1 vs 3	Day 2 vs 3				Day 1 vs 2	Day 1 vs 3	Day 2 vs 3
Before multiple Imputation	3.08 (1.45-6.40)	2.39 (1.22-4.39)	2.24 (0.97-4.38)	4.09; p>0.99	4.58; p>0.99	0.49; p>0.99	3.84 (2.04-13.3)	3.0 (1.38-6.09)	1.79 (1.23-3.50)	7.34; p=0.39	12.06; p=0.049	4.71; p>0.99
After multiple imputation 1	3.17 (1.46-6.65)	2.39 (1.34-4.08)	2.21 (0.65-4.12)	6.15; p=0.80	9.15; p=0.29	3.0; p>0.99	3.81 (2.04-12.93)	3.0 (1.38-6.09)	1.79 (1.16-2.99)	8.5; p=0.37	15.35; p=0.016	6.85; p=0.64
After multiple imputation 2	3.17 (1.46-6.37)	2.86 (1.47-4.89)	2.67 (1.84-4.38)	5.05; p>0.99	4.85; p>0.99	-0.20; p>0.99	3.81 (2.04-12.93)	1.69 (1.36-5.18)	1.42 (1.27-2.99)	10.55; p=0.17	16.0; p=0.011	5.45; p=0.97
After multiple imputation 3	3.17 (1.46-6.65)	1.76 (1.31-4.08)	2.67 (1.16-4.38)	9.05; p=0.30	5.5; p=0.96	-3.55; p>0.99	3.81 (2.30-12.93)	1.68 (1.15-5.18)	1.79 (1.27-4.33)	11.6; p=0.11	13.75; p=0.038	2.15; p>0.99
After multiple imputation 4	3.17 (1.46-6.65)	1.91 (0.97-4.08)	1.35 (0.73-3.33)	8.0; p=0.44	12.85; p=0.06	4.85; p>0.99	3.81 (1.83-12.93)	3.52 (1.42-6.09)	1.53 (0.84-2.99)	5.80; p=0.88	15.65; p=0.014	9.85; p=0.22
After multiple imputation 5	3.17 (1.46-6.65)	3.0 (1.42-5.14)	2.21 (0.97-4.12)	3.90; p>0.99	8.55; p=0.36	4.65; p>0.99	3.81 (1.83-12.93)	3.0 (1.38-6.09)	1.42 (1.27-2.58)	6.40; p=0.74	15.5; p=0.015	9.10; p=0.30

*Dunn's multiple comparison test expressed by mean rank difference and p-value. MTH, Mild therapeutic hypothermia. Gal-3, galectin-3. IQR, interquartile range.
